# Supplementary material for: The perpetual fragility of creeping hillslopes
Source: Nat Commun. 2021 Jun 23;12:3909. doi: 10.1038/s41467-021-23979-z (PMC8222271; doi:10.1038/s41467-021-23979-z)
Supplement: Supplementary file 1 — Supplementary Information [file 41467_2021_23979_MOESM1_ESM.pdf]

Supplementary materials for: The perpetual fragility of creeping hillslopes

Nakul S. Deshpande[1], David J. Furbish[2,3], Paulo E. Arratia[4], and Douglas J. Jerolmack[1,4]

[1]Department of Earth and Environmental Science, University of Pennsylvania, Philadelphia, Pennsylvania, USA
[2]Departments of Earth and Environmental Sciences Vanderbilt University, Nashville, Tennessee, USA
[3]Civil and Environmental Engineering, Vanderbilt University, Nashville, Tennessee, USA
[4]Department of Mechanical Engineering & Applied Mechanics, University of Pennsylvania, Philadelphia, Pennsylvania, USA

April 26, 2021

Abstract

In the main text, we describe the salient phenomenology of sub-yield granular creep experiments and a comparison with field measurements. Herein we present supplemental materials, including extended data figures and methods, that provide additional details helpful for understanding, validating, and reproducing our work and approach. We begin by revisiting the foundational theory of hillslope soil creep by Culling [1], in the context of modern granular mechanics. We then proceed to a reanalysis of data from the canonical experiments of disturbance-driven hillslope creep by Roering et al. [2], where we demonstrate that the induced grain motions are in fact in the inertial (rather than creep) regime. Finally, we provide extended figures, methods, and phenomenology that complement those reported in the main text.

***Keywords*—** geomorphology, granular physics, glassy dynamics, relaxation and rejuvenation, aging

Contents

Supplementary Note 1:
A Physical Interpretation of Culling's Classic 1963 Formulation of Soil Creep **3**

Supplementary Note 2: Reanalysis of Roering experiments **4**

Supplementary Figure 1: Reanalysis of canonical hillslope soil transport experiments 4

Supplementary Note 3: Extended experimental setup **5**

Supplementary Figure 2: Detailed optical and experimental setup 5

Supplementary Note 4: Materials **6**

Supplementary Figure 3: Creep phenomenology for different materials 7

Supplementary Note 5: Determining Strain and Velocity Profiles **8**

Supplementary Figure 4:
Determining Experimental Strain Profiles from Diffusing Wave Spectroscopy (DWS) 8

Supplementary Figure 5: Experimental strain-rate profiles 9

Supplementary Figure 6: Profile compilation 10

Supplementary Table 1: Fit parameters 10

Supplementary Note 6: Temperature and Humidity Effects **11**

Supplementary Figure 7: Ambient temperature and humidity conditions (February 12th, 2020) 11

Supplementary Figure 8: Long-duration (11 day) creep experiment 12

Supplementary Note 7: Extended disturbance phenomenology **13**

Supplementary Figure 9: Measured heat and acceleration 13

Supplementary Figure 10: Heating perturbation experiment: correlation functions 14

Supplementary Figure 11: Cyclic heating 15

Supplementary Figure 12: Sustained tapping and the development of a yield surface 16

Supplenentary Figure 13: Aftermath of extended tapping 17

Supplementary Note 8: Spatial correlation of the strain field **18**

Supplementary Figure 14: Quadrupoles at three times 18

Supplementary Note 1:
A Physical Interpretation of Culling's Classic 1963 Formulation of Soil Creep

Introduction and physical interpretation

W. E. H. Culling's 1963 paper on soil creep [1], published in *The Journal of Geology*, was a deeply insightful contribution 40 years ahead of its time. This paper is perhaps the most cited example of a theoretical formulation for slope-dependent soil transport. Yet elements of the formulation, drawn mostly from kinetic theory and statistical mechanics, remain unfamiliar. Here we unfold the formulation in practical terms. Focusing on the how-it-works part of the paper, here are technical elements. Culling envisioned soil on a hillslope as a rather special granular gas. He started with the Einstein-Smoluchowski [3, 4] description of the behavior of a Brownian particle, then appealed to the Master equation as elaborated by Chandrasekhar [5] to generalize Brownian motion to the idea of diffusion of many particles. Culling argued that for this special gas, particle motions are limited by the availability of space (porosity). Recognizing that particles are at rest most of the time, he appealed to Jeans's [6] kinetic formulation of the mean free path to estimate the magnitude of the diffusivity D — which is **not** the same as the diffusivity currently used in models of hillslope evolution. Culling also recognized the significance of soil production, but did not elaborate this idea. A key element of the formulation is the presence of a density stratification (i.e., porosity declines exponentially with depth), with surface-normal (upward) diffusion balanced by gravitational effects. In turn, a resulting horizontal concentration gradient leads to a downslope diffusive flux.

Now, here is a physical interpretation. Envision a rigid airtight shoebox full of air molecules. The mean free path is about $\lambda \sim 10^{-7}$ m, but this is still $O(10^3)$ larger than the molecular diameter d yielding a porosity $\phi \sim 1$. To a good approximation the motion of each molecule between successive collisions is a straight line; but in actuality it is parabolic in the gravitational field, and the gas therefore is density stratified. Imagine in a Harry Potterish [7] way turning the molecules into massive grains, then cool the gas to about 10 K so that it mostly "settles" to the bottom of the box, becomes dense ($\lambda \sim d$), and develops the resemblance of a free surface. Now one can actually see the parabolic trajectories, and importantly, the gas remains density/porosity stratified. Add a slight surface stickiness to the grains so that many/most are at rest most of the time, and replace their conserved thermal agitation with dissipative activations due to space creating micro-disturbances (left to the imagination). Tip the box, and add cooled (sticky) grains to the bottom, the analogue of soil production. The gas sort of flows, but strictly speaking, it is not a fluid; the bulk motion still results from the quasi-random stuck-then-unstuck space-limited diffusive motions of the grains. This is Culling's soil creep.[1]

Summary of the theory

Culling [8] had previously proposed that the granular flux q under the influence of gravity is described by a Fickian-like relation involving the land-surface slope, $q = -K\partial\zeta/\partial x$, which, when substituted into a statement of conservation leads to a diffusion equation, $\partial\zeta/\partial t = K\partial^2\zeta/\partial x^2$, of land-surface elevation ζ. To justify this result, Culling [1] then followed the analysis of Einstein [3] and envisioned local Brownian-like particle displacements l involving the diffusivity $D \sim n\langle l^2\rangle$ with number displacement frequency n. He then appealed to a master equation describing changes in particle number density [5] and performed an approximate vertical integration over the soil column to obtain the desired result. The formulation effectively envisions a slowly diffusing dense granular gas giving a bulk downslope motion due to the influence of gravity.

[1] Basis of [abbreviated] abstract presented by D. J. Furbish at AGU Fall Meeting 2017

Supplementary Note 2: Reanalysis of Roering experiments

Canonical sandpile experiments conducted by Roering and colleagues [2] remain the touchstone work in attempts to study hillslope soil creep in the laboratory. In this system, acoustic noise (meant as a representation of the myriad of disturbance types) was applied to one sidewall of a wedge of sand at two intensity settings, and grain velocities were measured (Supplementary Figure 1a). The resulting grain motions were described as creep in the original work. In the context of granular rheology, flows can be classified via a dimensionless shear rate known as the inertial number: $I \equiv \dot{\epsilon} d / \sqrt{P/\rho}$, where $\dot{\epsilon}$ is shear rate, d is grain size, P is confining pressure and ρ is density [9]. The physical meaning of the inertial number can be conceptualized as the ratio of two timescales: the inertial timescale of grain rearrangement $(d/\sqrt{P/\rho})$ and the time of macroscopic strain $(1/\dot{\epsilon})$. Three generic regimes of granular rheology have been identified based on I: (i) collisional or ballistic $(I > 10^{-1})$ which corresponds to a granular gas; (ii) dense and viscous-like flow corresponding to a hydrodynamic phase $(10^{-3} < I < 10^{-1})$; and (iii) quasi-static or solid-like deformation $(I < 10^{-3})$. More recently, it has been found that creep occurs for $I < 10^{-5}$ ([10]). Here we recast the results of Roering [2] in the context of the inertial number. We digitized the data reported in Figure 1B in Roering [2] using Web Plot Digitizer, and calculated the inertial number as a function of depth (Supplementary Figure 1). For a granular heap, the confining pressure increases with depth (z) due to the hydrostatic burden of the grains above: $P = P_0 + \rho g z \phi$, where $\rho = 2.65 g/cm^3$ is the assumed grain density, g is gravity and $\phi = 0.6$ is the assumed packing fraction of the grains. The term P_0 accounts for the weight per unit area of grains on the surface (i.e., at $z = 0$) and is defined as $P_0 = \alpha(\rho g d)$, where $\alpha = 0.1$ is an empirically-derived constant [10]. Our re-analysis demonstrates that measured grain motions in these experiments were not in the creep regime: the 'creeping' surface layer described in the original paper sits well within the dense-granular flow regime, and indeed all resolved motions down to the deepest depths are significantly above the transition to creep (Supplementary Figure 1b). This indicates that the imposed acoustic noise effectively fluidized the grains. Finally, these inertial numbers are three orders of magnitude larger than the ones we measure in our experiments (see Supplementary Figure 5).

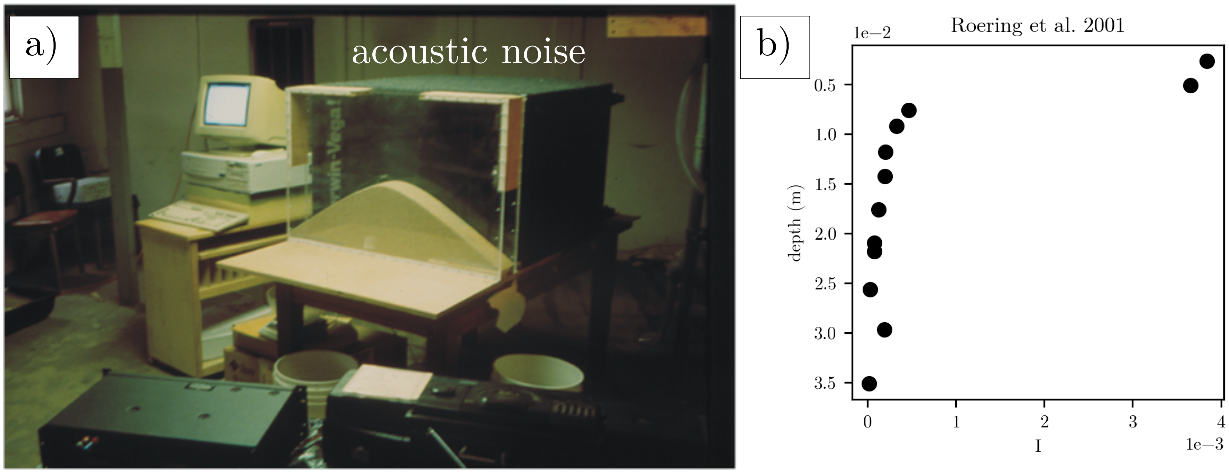

Supplementary Figure 1: Reanalysis of the canonical hillslope soil transport experiments. a) Experimental setup of Roering and colleagues [2] - which is similar to our system geometry, reported in the main text. b) Reanalysis of velocity profile reported in Figure 1B in the original work [2], in terms of the Inertial number. Data indicate that all measured grain velocities were in the dense granular flow, rather than creep, regime. It seems certain that grains far below the surface would have been creeping, however measurements of slow moving grains were not taken.

Supplementary Note 3: Extended experimental setup

Using a XIMEA CCD camera (model MD028MU-SY) with 1456x1936 pixels, we begin by acquiring images of an empty cell. The cell is illuminated with a laser beam ($\lambda = 633$ nm, 5.0 mW), which we pass through an expanding lens, a holographic diffuser, and a collimating lens (Supplementary Figure 2). This method follows other studies that measure spatially-resolved dynamics in soft materials with laser speckle imaging [11, 12] and allows us to render a coherent laser spot that illuminates much of the sandpile. The dimensions of the expanded beam are such that most of the light intensity is focused within a 7x7-cm region. The camera is placed about 50 cm away from the sidewall of the heap. The magnification is 3.6 x 10^{-5}m/px, or about 3.6 pixels per grain diameter. To achieve spatially-resolved measurements, we partition the images into grids the size of l^*, the mean free path of light in the material, which is about three times the grain diameter ($= 3.3d$). Therefore, we set each metapixel size to be 10 pixels. The quantity of interest is the intensity correlation function:

$$G(t, t+\tau) = \frac{\langle I_t I_{t+\tau}\rangle - \langle I_t\rangle \langle I_{t+\tau}\rangle}{\sqrt{\langle I_t^2\rangle - \langle I_t\rangle^2}\sqrt{\langle I_{t+\tau}^2\rangle - \langle I_{t+\tau}\rangle^2}}$$

where I indicates the metapixel intensity, $\langle\rangle$ are spatial averages within a metapixel and c is a constant which depends on the experimental parameters: $c = 8\pi\frac{\sqrt{2/5l^*}}{\lambda}$, where λ is the wavelength of light. Thus, G is a quantity which indicates the change in the speckle pattern within a metapixel between a start time t and a lag time τ. Using optical theory [13], we can map G to a physical quantity: ε, the amount of strain which occurs inside a volume fixed by l^*: $G(t,\tau) = exp(-c(\bar{\varepsilon} + \bar{\zeta}))$. There are two contributions to changes in G: ε is the strain and ζ are random motions. We note that strain here is agnostic about the precise nature of grain motions; in other studies where the system is driven by shear flow, motion is decomposed into contributions due to pure shear [14]. In our images, we calculate G for each metapixel, and report its ensemble-average across the whole pile, $\langle G\rangle$, when rendering the correlation function. We establish our noise floor by benchmarking the sensitivity of G, by acquiring speckle images of a static ensemble of grains enclosed within a sealed glass beaker with a flat surface slope. A laser beam is expanded and illuminates the beaker, and we calculate G following the same protocol used above. We observe none of the the phenomenology observed within our experiments (because grains in the beaker are not creeping), but the correlation decays from 1 to 0.9 over 1000 seconds; this suggests error of order 10 % in the globally-averaged G at long lag times.

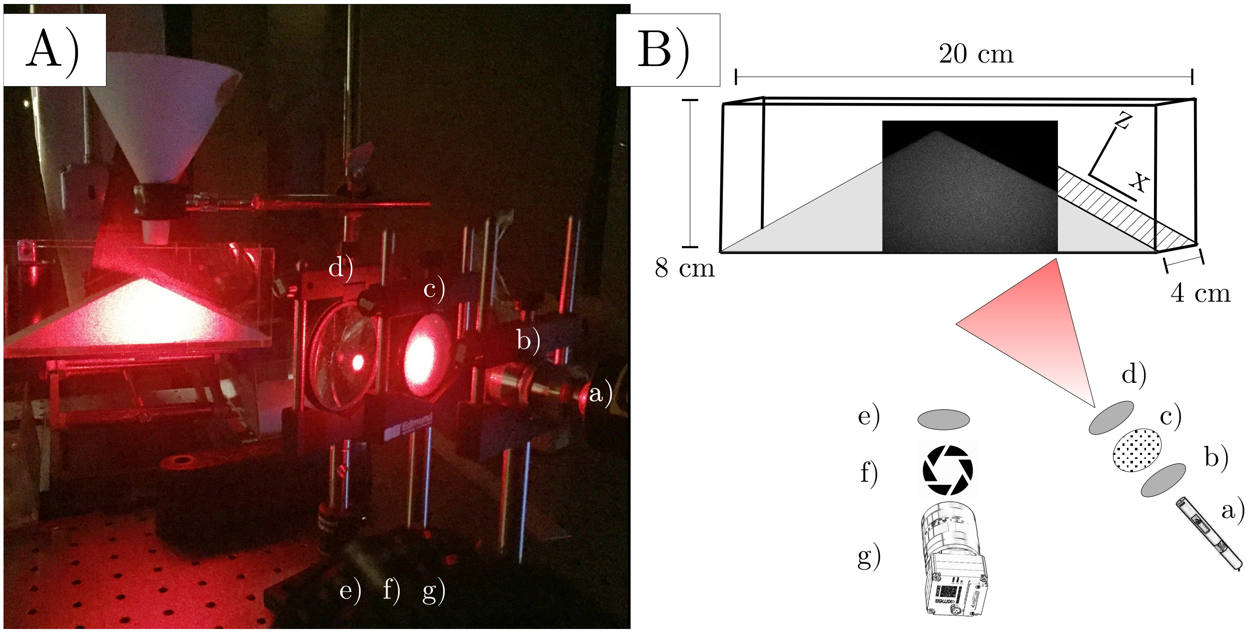

Supplementary Figure 2: Detailed optical and experimental setup. Components: a) HeNe laser ($\lambda = 633$ nm, 5.0 mW); b) expanding lens; c) holographic diffuser; d) collimating lens; e) circular polarizer; f) diaphram; and g) CCD camera. Entire setup rests on a vibration-isolating optical table Newport Integrity 4.

Supplementary Note 4: Materials

In the main text we report on experiments conducted using glass beads having uniform diameter $d = 100\mu m$. As described there, these glass beads were chosen because they have known optical properties, which allows us to compute strain ϵ from measured correlation functions $G(\tau)$. To test whether changing material properties had a significant effect on creep dynamics, we also examined three other materials: (i) polydisperse quartz 'playground' sand with a median diamter $d \approx 500\mu m$; (ii) a 50/50 mixture (by volume) of industrial kaolinite powder ($d \approx 10\mu m$) and the playground sand; and (iii) pure kaolinite powder. The optical and scattering properties of these materials are not known, and so strain rates determined using parameters for the glass beads introduce unknown errors with these other materials; nevertheless, relative changes in strain rate within a given experiment should be reliable. We observe that dynamics of the playground sand are essentially the same as observed for glass beads (Supplementary Figure 3); strain is spatially heterogeneous but diminishes with depth, $x-$averaged strain profiles show an exponential decline with depth and correlations decay systematically with increasing start times t. Kaolinite introduces significant heterogeneity to the grain motions: while strain activity still diminishes on average with depth, large-scale coherent zones of strain are sufficiently extensive that strain profiles fluctuate with depth (rather than monotonically decrease). Nevertheless, the qualitative dynamics are similar. Moreover, we speculate that larger horizontal ($x-$) averaging on a larger pile would likely smooth over these fluctuations to produce an exponential strain profile, though observations are needed to confirm this. Kaolinite likely introduces more significant relative humidity effects; exploring the role of compositional variation on the rates and styles of creep seems a fruitful avenue for future research.

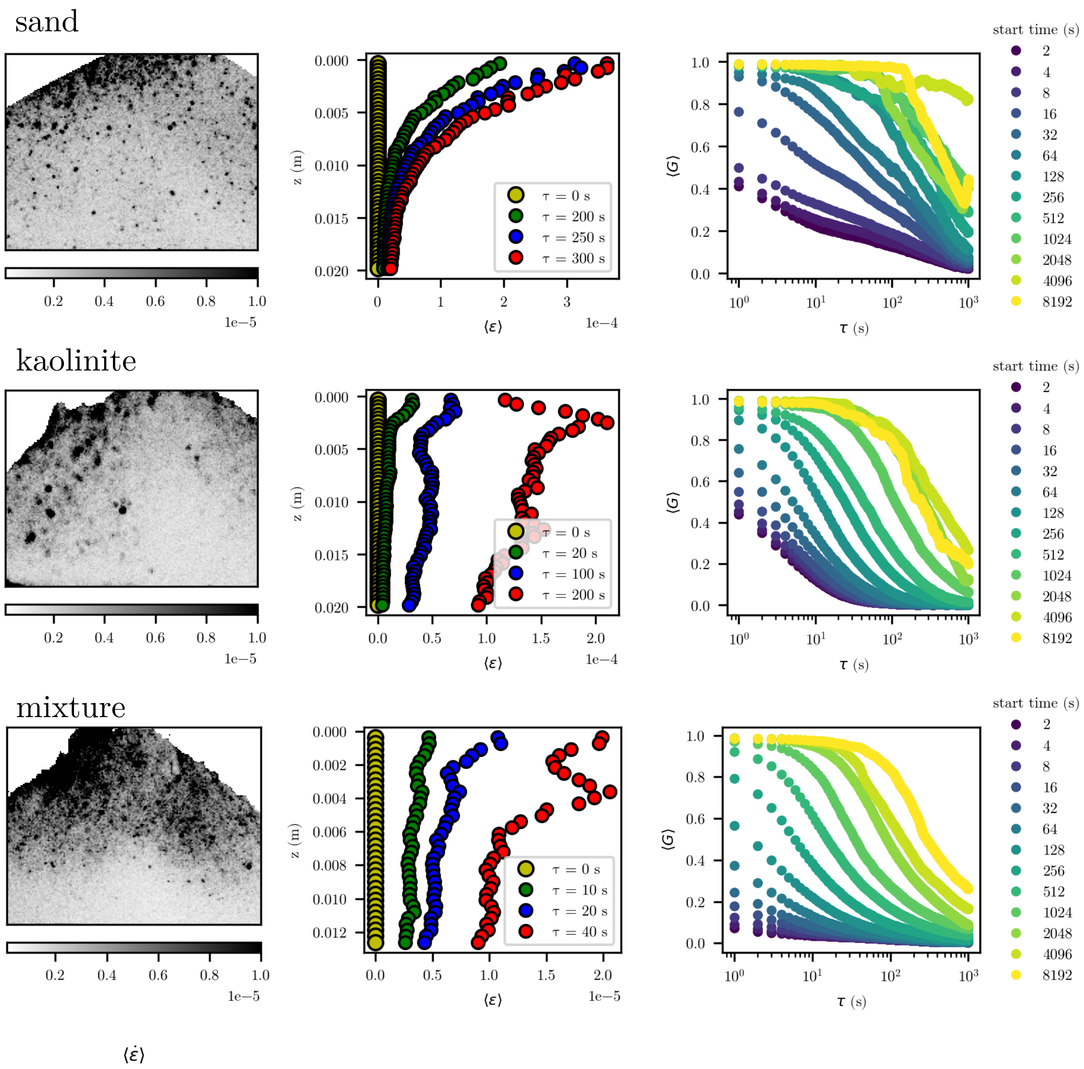

Supplementary Figure 3: Creep phenomenology for different materials. Maps of instantaneous creep deformation (left), example cumulative strain profiles (middle), and relaxation curves (right) for: rough playground sand (top); kaolinite powder (middle); and an equal mixture of both. Note the differences in the spatial heterogeneity in the creep response between samples, and relatively larger zones of deformation in the kaolinite sample.

Supplementary Note 5: Determining Strain and Velocity Profiles

Here we report the procedures for producing depth-resolved profiles of strain for our laboratory experiments, and also for determining velocity profiles of creeping hillslope soils from previously published work.

Determining Experimental Strain Profiles from Diffusing Wave Spectroscopy (DWS)

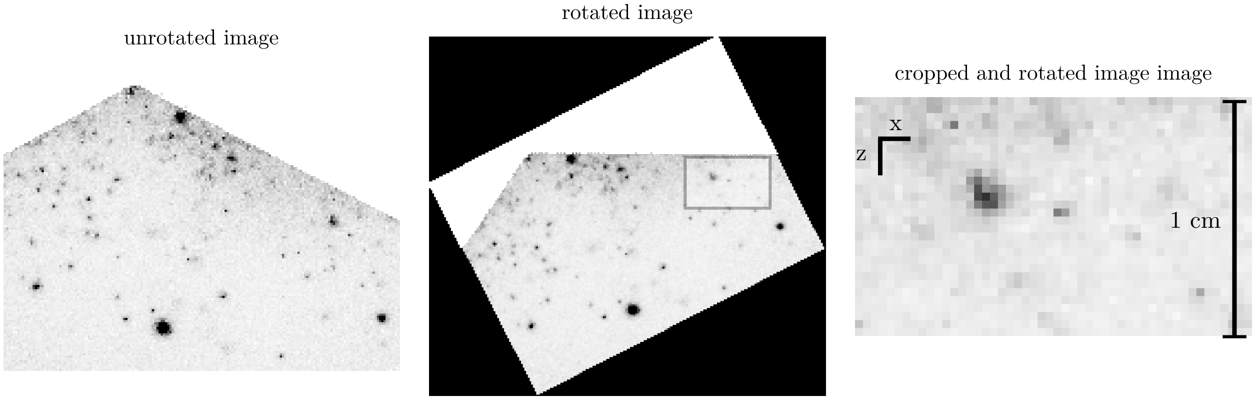

Supplementary Figure 4: Experimental DWS profile protocol. Snapshot of spatially-resolved strain (left) for an arbitrary image from an undisturbed creep experiment, taken at $t = 2048s$ and lag time $\tau = 20$ s. Depth-resolved strain profiles are measured from a region of interest (50 x 30 pixels, middle panel), where depth is determined normal to the slope surface. We chose a region of interest as far as possible from the pile apex, because we expect that the latter is most sensitive to the initial conditions. Average strain values per unit depth are calculated by horizontal (x) averaging of pixels within a fixed depth (z) interval (right panel). For future comparison with field profiles, these experimental data are fit with an exponential decay of the form $\dot{\epsilon}/\dot{\epsilon}_0 = e^{-z/\lambda}$, where λ and $\dot{\epsilon}_0$ were determined using a least-squares minimization routine in the Python library scipy.

Maximum strain-rates, inertial numbers and constraining velocities

Although exceedingly sensitive to grain motions, strains extracted from DWS are somewhat ambiguous; G encapsulates both affine and non-affine deformations [13, 15] — this obfuscates translating strain into net downslope transport rates. With extended DWS experiments, deeper leveraging of light scattering theory or a known direction of forcing, these components can be deconvolved [16, 17]. Nonetheless, here we constrain measured strain-rates such that they can be compared to field measurements by considering two end-member cases: i) all strain is shear (downslope transport) and ii) all strain is gravitationally-driven compaction. Our fastest measured strain rates, at the beginning of an experiment when the sandpile is at its least compacted state, are of order $\dot{\epsilon} = 10^{-5}\frac{1}{s}$ (Supplementary Figure 5). If all strain is affine (horizontal direction, simple shear), then for all grains contained in a metapixel, the average velocity is $u = \dot{\epsilon}l^*$ (recall that $l^* = 3d$) and we obtain $u \approx 3$ x $10^{-9}\frac{m}{s}$, or $0.1\frac{m}{yr}$. Note that this velocity is of the same magnitude as the fastest creeping soils included in our data compilation (Supplementary Figure 6). Now consider that this reference frame is on a slope. If all of the velocity is in the downslope direction then this component is: $usin\theta = 0.05\frac{m}{yr}$. If instead all strain goes into compaction, this component is $ucos\theta = 0.09\frac{m}{yr}$, where $\theta = 30$, the angle of repose. Thus, the magnitude (not just the functional form) of our measured creep rates are comparable with those measured in field data (Supplementary Figure 6). These maximum strain-rates also constrain the maximum inertial number. Recall that the inertial number is: $I \equiv \dot{\epsilon}d/\sqrt{P/\rho}$, where $\dot{\epsilon}$ is shear rate, d is grain size, P is confining pressure and ρ is density [9]. For grains near the free surface, the maximum measured strain-rates are of the order $\dot{\epsilon} = 10^{-5}\frac{1}{s}$, the pressure is heavily influenced by the term P_0 and the maximum Inertial numbers are $\approx O(10^{-6})$ – well within the quasi-static creeping regime [10].

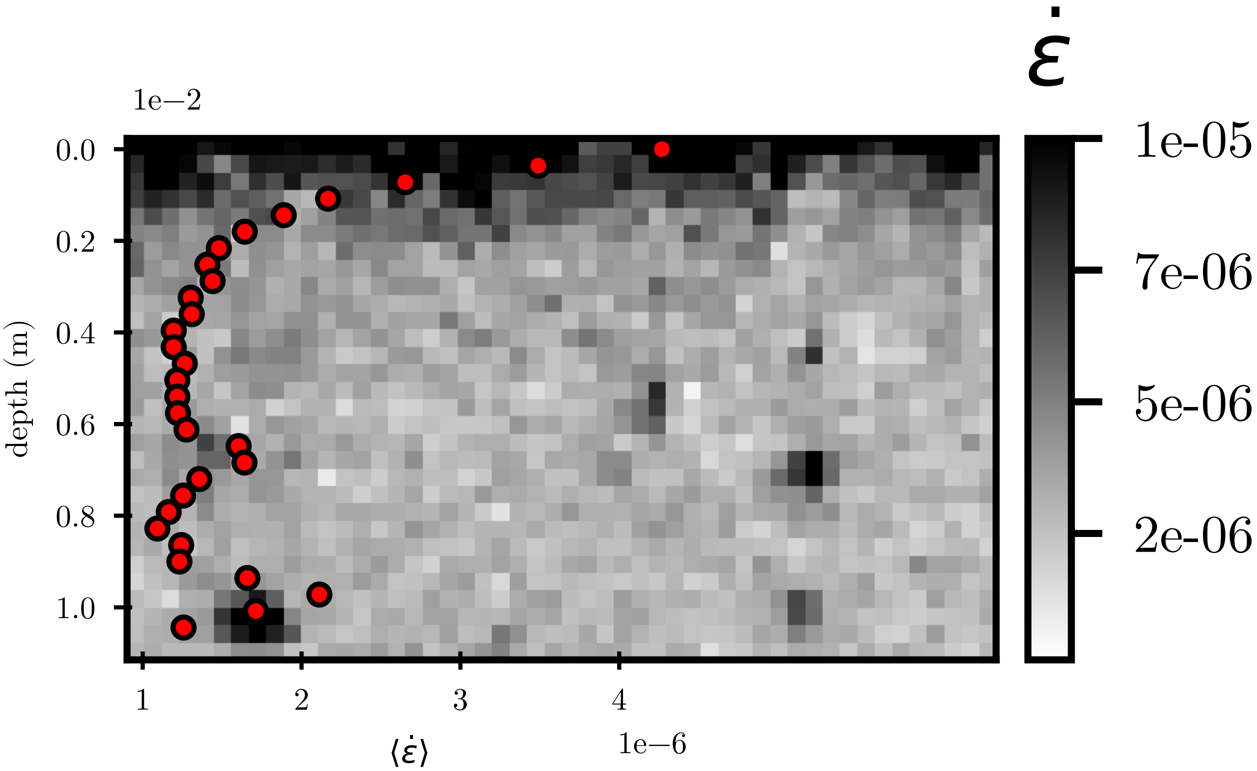

Supplementary Figure 5: Experimental strain-rate profiles of sandpile at t = 2s. The initial strain profile measured from DWS experiments. Note that the inertial numbers corresponding to these experiments are well within the quasi-static creeping regime, and are $O(10^3)$ smaller than those calculated from the Roering experiment. Red dots indicate horizontally-averaged instantaneous strain-rates.

Hillslope soil creep rate profiles from the field, determined from Young Pits

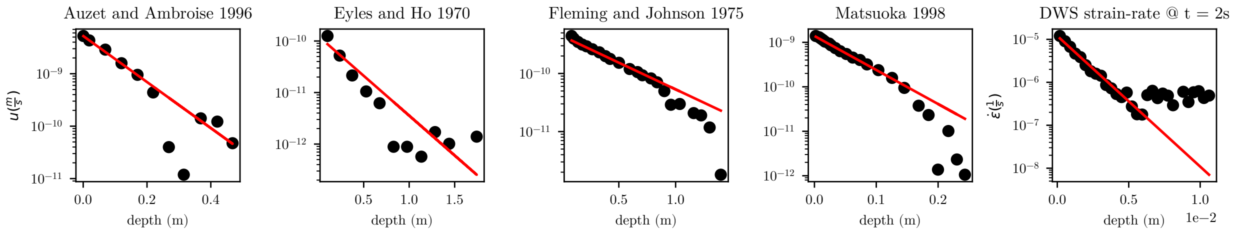

Supplementary Figure 6: Profile compilation. Four hillslope soil creep velocity profiles compiled from the literature [18–21]. The last panel shows a measured strain-rate profile from one of our experiments determined from DWS; it is obtained by dividing each cumulative strain profile by the time elapsed from the start time. Experimental data are shown for $t = 2$ s, just after preparation of the pile, when creep rates are fastest and the pile has not 'aged'. Field data were digitized from a previous study [22], which qualitatively compared these profiles with those measured in acoustically-driven flows. We excluded two profiles cited in that paper — an experimental work on solifluction [23] and a measurement of glacial till deformation [24]. We chose to do this because our purpose was to compare data from field studies of hillslope soil creep only. As noted before [22], concave-down, exponential-like velocity profiles are not observed in all field measurements; irregular profile shapes are sometimes seen [25]. We speculate that in these environments, macro-scale disturbances are generated by large elements such as tree throw, or heterogeneity in groundwater hydrology and/or soil properties. Nevertheless, profiles in the reported four field studies are consistent with an exponential fit $u/u_0 = e^{-z/\lambda}$ (shown in red), which we obtain by performing a least-squares regression. Note that for the DWS profile there is a sharp deviation from the exponential decay at $\approx 0.005m$ depth, where strain rate seems to saturate. We speculate that at early start times, when the pile is at its least compacted state and the free surface begins to creep, that this creep slowly propagates downward into the pile as a front. At this initial stage, deep enough grains have not yet been reached by this propagating front. At later start times, depth profiles become more developed and this deviation goes away, but the profile remains exponential.

Study	$u_0(\frac{m}{s})$	$\lambda(m)$	R^2
Auzet and Ambroise (1996)	5.25 x 10^{-9}	0.10	0.99
Eyles and Ho (1970)	1.25 x 10^{-10}	0.28	0.88
Fleming and Johnson (1975)	4.42 x 10^{-10}	0.47	0.97
Matsuoka (1998)	1.39 x 10^{-9}	0.06	0.99
	$\dot{\epsilon}_0(s^{-1})$		
DWS profile	1.20 x 10^{-5}	0.001	0.97

Supplementary Table 1: Maximum velocities, strain-rate, coefficient λ and correlation coefficient R^2 determined from fitting the function $u/u_0 = e^{-z/\lambda}$ to depth profiles of for field data) and $\dot{\epsilon}/\dot{\epsilon}_0 = e^{-z/\lambda}$ to experimental DWS data.

Supplementary Note 6: Temperature and Humidity Effects

For the experiments reported in the main text, temperature and relative humidity remained approximately constant over the duration of each experiment. An example is shown below (Supplementary Figure 7).

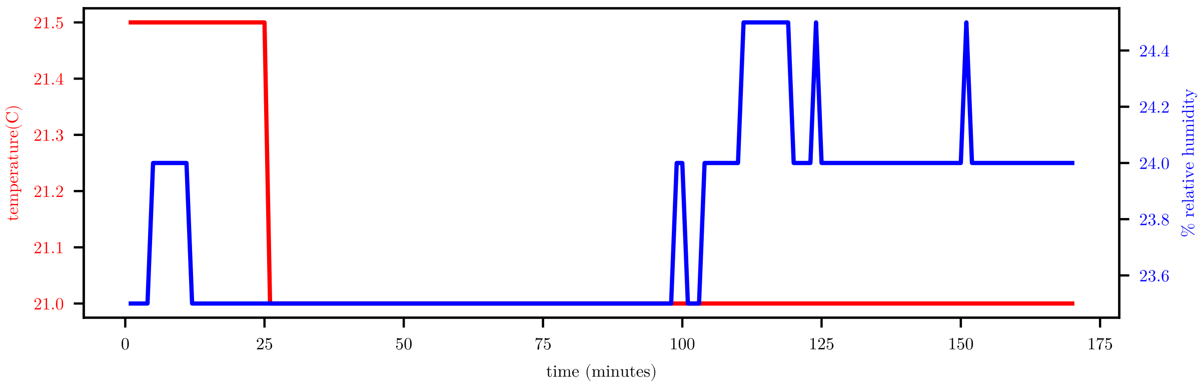

Supplementary Figure 7: Ambient temperature and humidity conditions (February 12th, 2020). Data shown here for a representative experimental run, corresponding to Figure 2 in the main text. Ambient temperature (21C +/- 0.2) and relative humidity (23.8% + 0.3) were fairly constant over the experimental duration. The variables were recorded with a USB environmental logger (Lascar) at a frequency of 1 HZ. Discrete jumps show the resolution of the measurements. All experiments reported in the main text were conducted under comparable, relatively constant conditions; so we interpret creep dynamics to be unaffected by environmental changes in temperature and relative humidity.

Long-duration experiments

An 11-day long creep experiment, with no prescribed disturbances, was run to examine creep for a longer duration than previous runs. However, over this period there were significant environmental changes, in terms of temperature and relative humidity (RH), that did not occur in shorter duration runs. These changes affected creep rates and styles, the results of which are reported in this section. Over the 11-day duration, temperature and RH experienced two, several-day cycles of up-and-down fluctuations (Supplementary Figure 8, top). The resultant creep dynamics were complex (Supplementary Figure 8, bottom): correlation functions were more erratic than observed for constant environment (and shorter-duration) creep experiments. Moreover, in contrast to constant-environment experiments — where relaxation timescales monotonically increased with time — we observed both increases and decreases in relaxation timescales from the start of the experiment to its end at 11 days (Supplementary Figure 8). This indicates that some aspect of the changes in temperature and RH acted to rejuvenate the pile, reversing aging and leading to some increases in creep rates. We suspect that most of these dynamics were the result of RH rather than temperature, because variations in the latter were relatively small. Future work should seek to directly control and measure the influence of humidity on creep phenomenology.

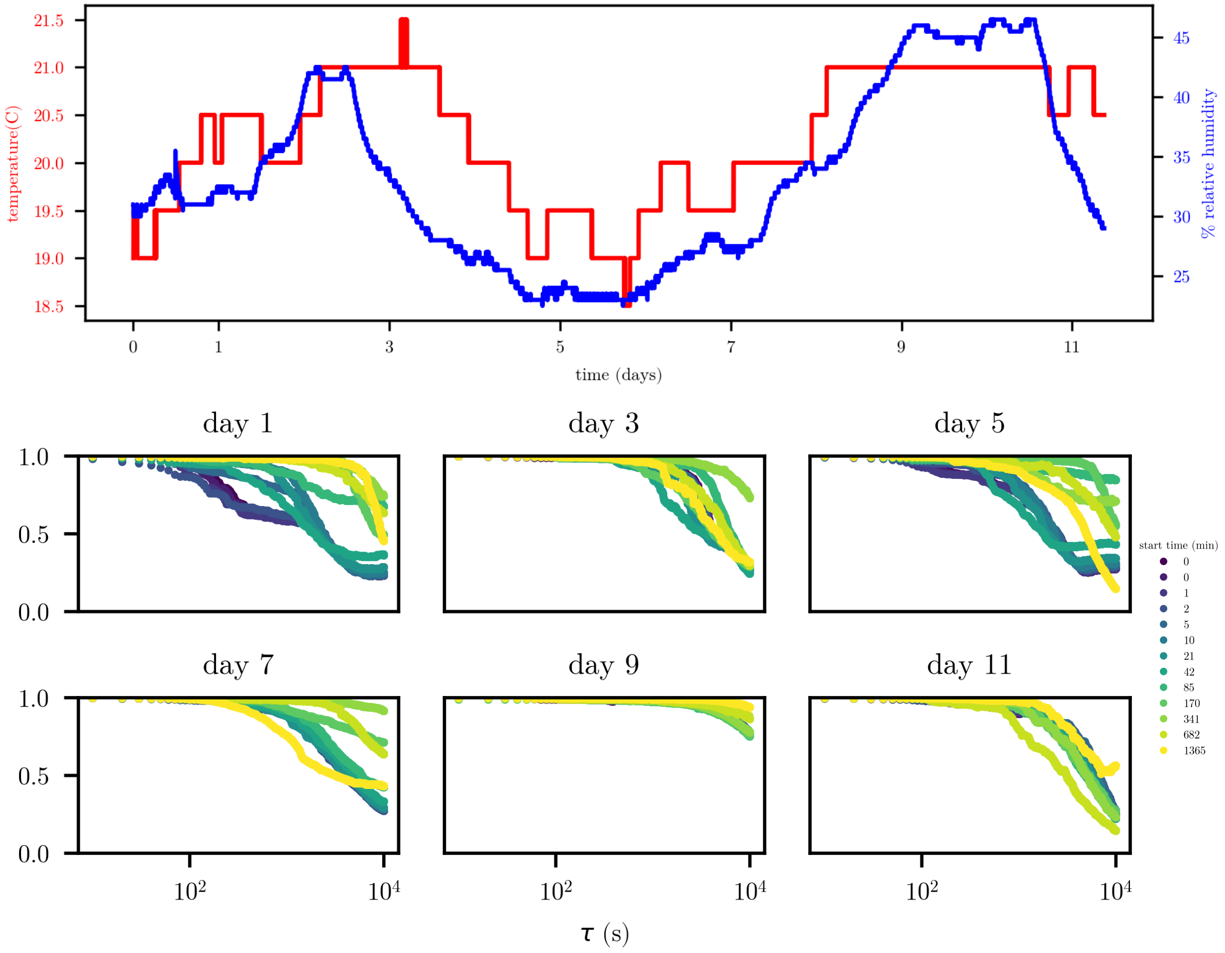

Supplementary Figure 8: Long-duration (11 day) creep experiment. A pile of grains was prepared using the same protocol outlined in the main text. Here, we provide extended observations of creep beyond the post-preparation period, and collect speckle images every other day since preparation. Each correlation function is composed of 10^4 s of observation, and start times are arbitrarily chosen to grow as $log_2 t$. The creep response is complex, and not clearly coupled to environmental fluctuations - although the slowest and most uniform decorrelations occurred during day 9, where the highest and most sustained relative humidity was recorded.

Supplementary Note 7: Extended disturbance phenomenology

Measuring heat and tapping

Here we provide additional experimental details for experiments involving disturbed creep. First, we report temperature readings at the sidewall of the cell for a heating disturbance experiment, and accelerations experienced by the pile as a result of tapping by the metronome for tapping disturbance experiments (Supplementary Figure 9).

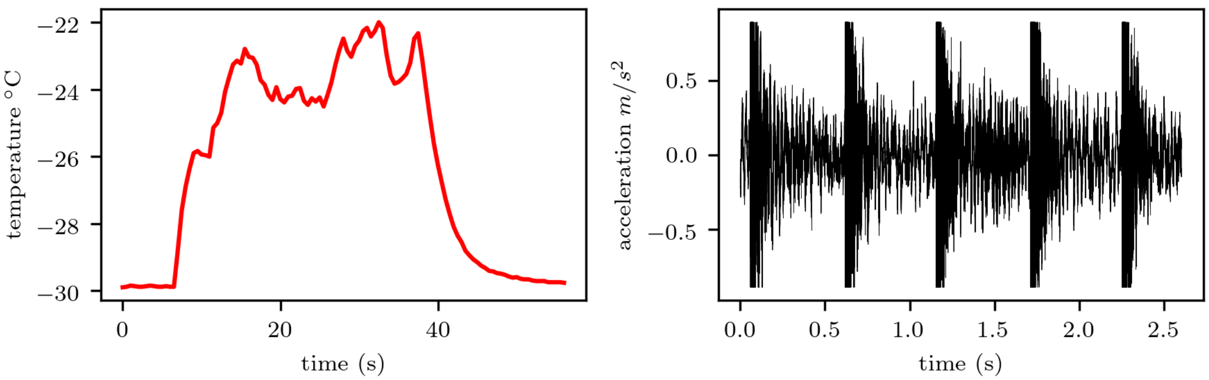

Supplementary Figure 9: Measured heat and acceleration. Left) Raw temperature readout from a thermistor at the wall of the experimental cell, during the heating experiment reported in Figure 3 of the main text. Heat was applied at $t = 10$ s and removed at $t = 40$ s; temperature oscillated between 50 C and 60 C during heat application, and then decayed to room temperature after heat was removed. Right) Acceleration measurements of 5 taps from the metronome; this represents the accelerations delivered to the pile for disturbed tapping experiments such as those reported in Figure 3 of the main text. Acceleration was measured with a Digiducer 333D01 at a 48-kHz sample rate.

Extended heat perturbation analysis

We next present the correlation functions associated with the heating disturbance experiments reported in Figure 3 of the main text (Supplementary Figure 10). As reported in the main text, application of heat enhances the creep rates by more than a factor of ten. Upon removal of heat, the correlation functions are qualitatively distinct from those before heating (i.e., pure relaxation) and exhibit rapid decay— indicating fast creep rates. With increasing time (and cooling), however, creep rates slow down and the correlation functions approach those observed before heating was applied (Supplementary Figure 10).

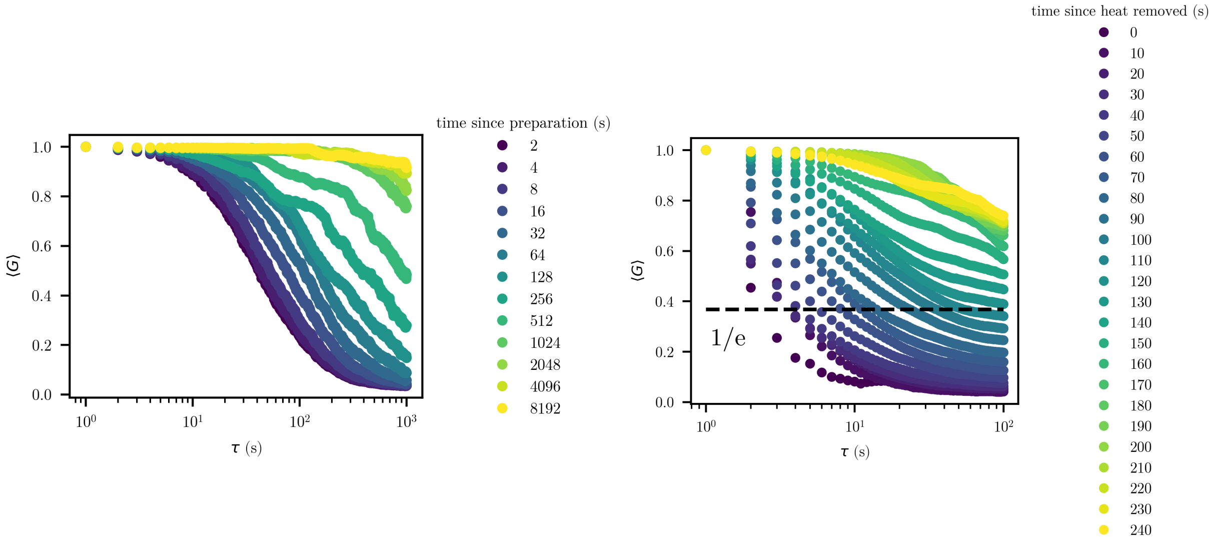

Supplementary Figure 10: Heating perturbation experiment: correlation functions. Left) Correlation functions for a purely relaxing pile, before heat is applied. Right) Correlation functions shown at 25 different times following removal of the heat source (data during heating are not shown). The relaxation timescales reported in Figure 3 in the main text were determined by the time at which the normalized correlation function reaches the value 1/e.

We conducted an additional experiment in which cyclic heating — rather than a single, 10-s application of heat as described previously — was applied, in order to examine whether long-term creep rates were altered by thermal cycling. Spatially-averaged creep rates were determined using the same methods described in the main text. In these cyclic heating experiments, heat was applied for 30 s and then removed, and then the pile was allowed to relax (and cool) for 200s before heat was applied again. Creep rates increased rapidly with heat application and declined gradually after removal — just as in the previous experiment. However, repeated thermal cycling produced a pattern in which creep rates fluctuated, but the average remained approximately constant through time. This is in contrast to pure relaxation experiments reported in the main text (Figure 3), where creep rates declined monotonically with time. These results imply that heating cycles can sustain a creep rate that does not decline with time. This result suggests that more work on how thermal cycles stress the grains is needed.

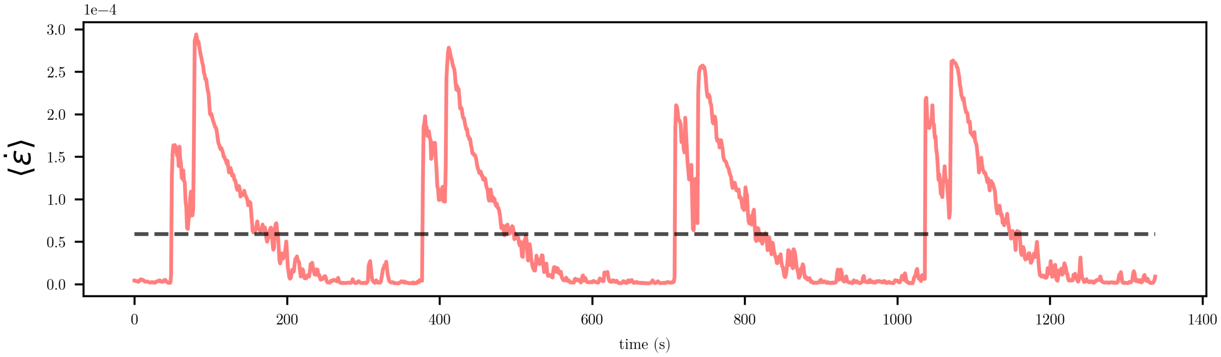

Supplementary Figure 11: Cyclic heating. Instantaneous creep rates during and following four cycles of applied heat. Black dashed line indicates the mean rate.

Extended tapping phenomenology

In the main text we reported that tapping induced the formation of a flowing surface layer, which transitioned at depth to creep beneath (Figure 4). Here we show supporting data for that claim. In the experiment, taps were administered at a frequency of 1 Hz, and images to determine strain were also measured at 1 Hz. Images were phase-lagged from the taps, however, so that they were measured between taps. We computed profiles of strain rate, and the Inertial number, following each tap, using the methods described earlier (Supplementary Figure 12). Indeed, the data show a fast surface layer developed that was approximately 4-mm deep, with a kink in the strain rate profile at its base below which the dynamics are slower and more heterogeneous. This bi-partite profile, with coherent fast motion in the surface layer and slower, heterogeneous motion beneath, has been reported previously in association with a transition from a dense granular flow to creep [10, 14, 26]. We compute depth-resolved profiles of the Inertial number and find, for the fastest surface layer, values approaching $I \sim 10^{-6}$. Experiments [10] and simulations [26] found, for steady shear flows, the creep-flow transition occurred for $I \sim 10^{-5}$. While our number is smaller than that, it is important to note that strain rates are computed from 1-s image differences, which integrates over a tap cycle (acceleration and relaxation). Note that accelerations from a single tap approach 1 m/s^2 for short durations ($<$ 0.1 s; Supplementary Figure 9); this implies that surface grains could briefly reach strain rates much higher than the 1-s averaged values. We thus believe that our measured value $I \sim 10^{-6}$ for the surface layer is a pronounced underestimate of the maximum instantaneous I, and that tapping temporarily excites a fluidized surface layer in which $I > 10^{-5}$.

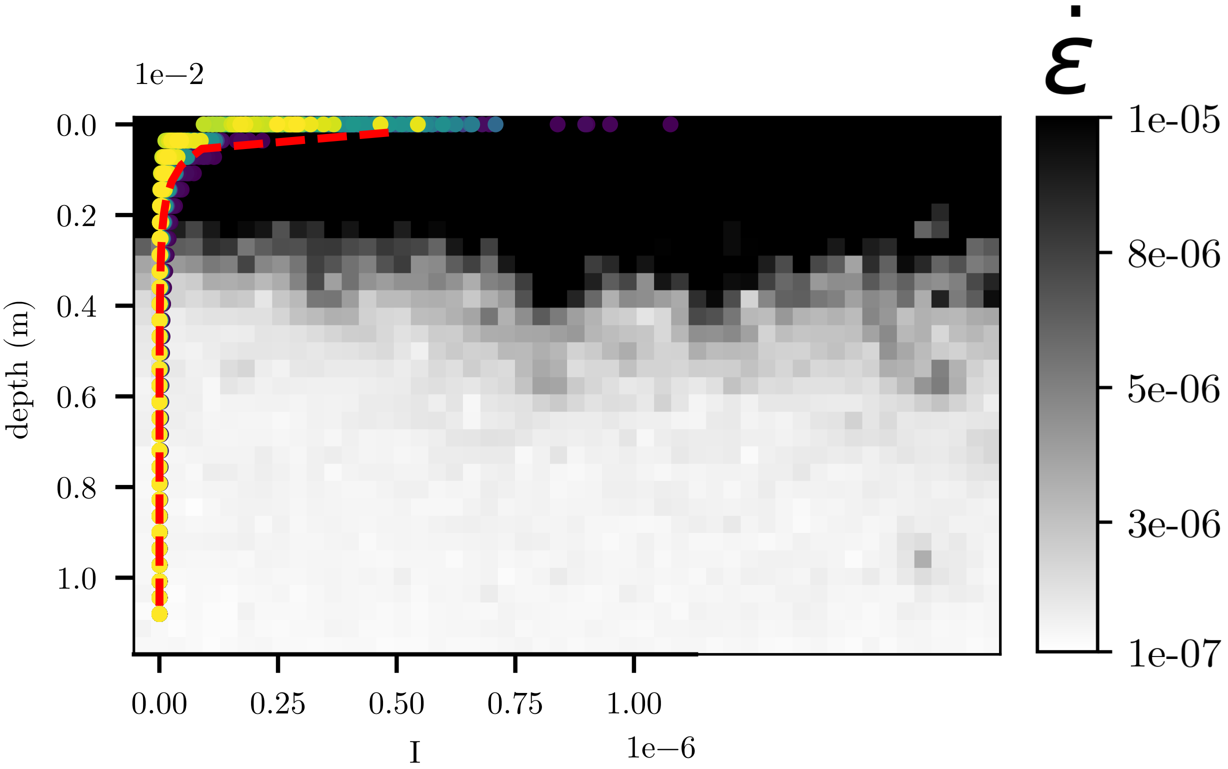

Supplementary Figure 12: Sustained tapping and the development of a yield surface. The qualitative picture from spatial maps (above and Fig. 3 in main text) is that the layer thickness appears to converge to a finite value within a few hundred taps. Here, different color dots represent different profiles in time; cool colors denote early taps and yellow colors are later taps. We note that most of the variability occurs above 0.004 m. We interpret this 4-mm surface layer as a dense-granular flow, and the region below it as creep. Similar behavior was reported in other systems exhibiting a flow to creep transition [10, 26]

Finally, we examine the 'aftermath' of tapping, in terms of creep dynamics following the application of 3000 taps (Supplementary Figure 13). This allows us to determine if and how tapping may have irreversibly affected the creeping of pile. The first thing to note is that, even through tapping significantly decreases overall creep rates (likely by compaction; main text, Fig. 3), nonetheless creep persists after tapping. The nature of the correlation functions, however, is qualitatively different from those observed for pure relaxation experiments. In particular, we observed quasi-periodic fluctuations that indicate some degree of reversible, elastic deformations (Supplementary Figure 13). The origins of this elastic regime are unknown. Future efforts should explore if and how disturbances such as tapping — which enhance rigidity by compaction, and the removal of ductility/plasticity — may lead to a more elastic behavior in granular heaps.

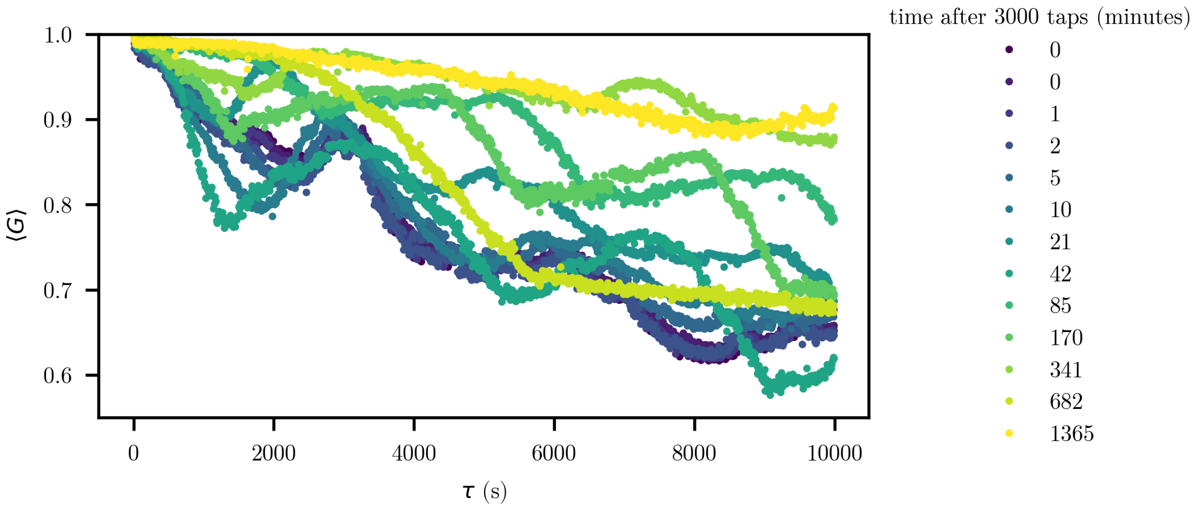

Supplementary Figure 13: Aftermath of extended tapping. Correlation functions of an undisturbed pile at 13 start times, following application of 3000 taps. Insofar as tapping compacts the sandpile and depletes susceptible zones of plastic, irreversible deformation, these data demonstrate that creep persists despite these effects. Note the quasi-periodicity of the correlations, which suggests some degree of reversible, elastic deformations. Future work should seek to reproduce and explore this elastic regime – and its connection with the elastoplastic modelling framework.

Supplementary Note 8: Spatial correlation of the strain field

In many athermal materials, there is a characteristic quadrupolar spatial signature of the dynamics that result when plastic flow events occur, and stress is locally redistributed [27–31]. To demonstrate that the decorrelations in our system are due to strain, and the connections between this strain and those other amorphous solids, we compute the spatial autocorrelation of the instantaneous strain-rate fields at three times (Supplementary Figure 14). The spatial autocorrelation function reads:

$$C_{\epsilon xy}(\Delta r) = \frac{\langle (\epsilon_{xy}(r + \Delta r) - \langle \epsilon_{xy} \rangle) \rangle \, (\epsilon_{xy}(r) - \langle \epsilon_{xy} \rangle)}{\chi^2}$$

Where ϵ_{xy} is the strain-rate at location xy, r is a distance away from xy and χ^2 is the variance [31]. Practically, we compute this by taking the 2D Fourier transform of a square region of interest of the strain-rate field, and then reversing the transform and dividing by the variance to normalize; this is the quantity C. Though this result is preliminary and the subject of future works, we include it here to emphasize the veracity of our mapping from G to ϵ and to point out further similarities between our system and the deformation and flow of other amorphous athermal materials. Exploring these similarities more rigorously is outside the scope of this work but we look forward to pursuing these questions in the future.

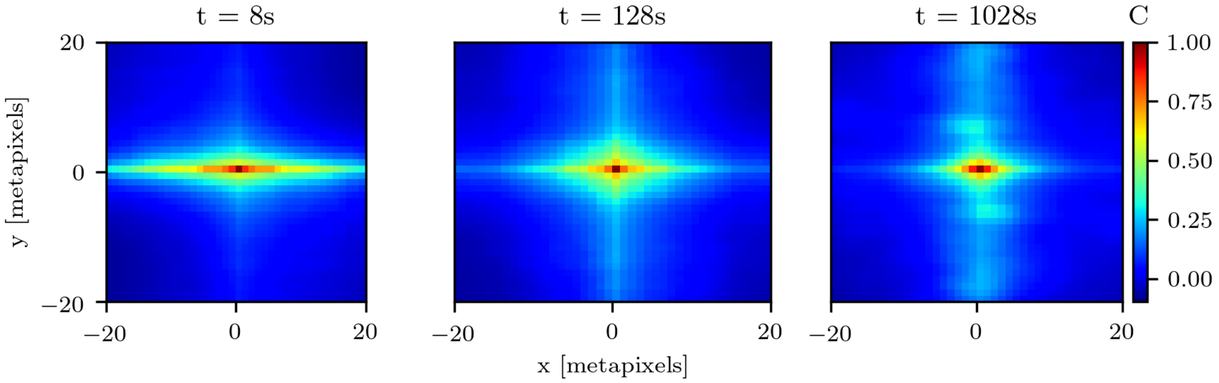

Supplementary Figure 14: Quadrupoles at three times. These are the 2d spatial autocorrelations for three different timesteps within our creep experiments. Note the symmetrical, quadrupolar shapes. The axes denote spatial coordinates in unites of metapixels.

Supplementary References

1. Culling, W. E. H. Soil Creep and the Development of Hillside Slopes. *The Journal of Geology* **71,** 127–161 (1963).
2. Roering, J. J., Kirchner, J. W., Sklar, L. S. & Dietrich, W. E. Hillslope Evolution by Nonlinear Creep and Landsliding: An Experimental Study. *Geology* **29,** 143–146 (2001).
3. Einstein, A. On the Motion Required by the Molecular Kinetic Theory of Heat of Small Particles Suspended in a Stationary Liquid. *Annalen der physik* **17,** 549–560 (1905).
4. Von Smoluchowski, M. Zur Kinetischen Theorie Der Brownschen Molekularbewegung Und Der Suspensionen. *Annalen der physik* **326,** 756–780 (1906).
5. Chandrasekhar, S. Stochastic Problems in Physics and Astronomy. *Reviews of modern physics* **15,** 1 (1943).
6. Jeans, J. *An Introduction to the Kinetic Theory of Gases* (CUP Archive, 1940).
7. Rowling, J. K. *Harry Potter and the Philosopher's Stone* (Bloomsbury Publishing, 2015).
8. Culling, W. E. H. Analytical Theory of Erosion. *The Journal of Geology* **68,** 336–344 (1960).
9. Jop, P., Forterre, Y. & Pouliquen, O. A Constitutive Law for Dense Granular Flows. *Nature* **441,** 727–730 (2006).
10. Houssais, M., Ortiz, C. P., Durian, D. J. & Jerolmack, D. J. Rheology of Sediment Transported by a Laminar Flow. *Physical Review E* **94,** 062609 (2016).
11. Scheffold, F. & Block, I. D. Rapid High Resolution Imaging of Diffusive Properties in Turbid Media. *Optics Express* **20,** 192 (2012).
12. Zakharov, P. & Scheffold, F. Monitoring Spatially Heterogeneous Dynamics in a Drying Colloidal Thin Film. *Soft Materials* **8,** 102–113 (2010).
13. Amon, A., Mikhailovskaya, A. & Crassous, J. Spatially Resolved Measurements of Micro-Deformations in Granular Materials Using Diffusing Wave Spectroscopy. *Review of Scientific Instruments* **88** (2017).
14. Katsuragi, H., Abate, A. R. & Durian, D. J. Jamming and Growth of Dynamical Heterogeneities versus Depth for Granular Heap Flow. *Soft Matter* **6,** 3023–3029 (2010).
15. Erpelding, M., Amon, A. & Crassous, J. Diffusive Wave Spectroscopy Applied to the Spatially Resolved Deformation of a Solid. *Physical Review E - Statistical, Nonlinear, and Soft Matter Physics* **78,** 1–9 (2008).
16. Crassous, J., Erpelding, M. & Amon, A. Diffusive Waves in a Dilating Scattering Medium. *Physical Review Letters* **103,** 1–4 (2009).
17. Aime, S., Ramos, L. & Cipelletti, L. Microscopic Dynamics and Failure Precursors of a Gel under Mechanical Load. *Proceedings of the National Academy of Sciences* **115,** 201717403 (2018).
18. Auzet, A.-V. & Ambroise, B. Soil Creep Dynamics, Soil Moisture and Temperature Conditions on a Forested Slope in the Granitic Vosges Mountains, France. *Earth Surface Processes and Landforms* **21,** 531–542 (1996).
19. Fleming, R. W. & Johnson, A. M. Rates of Seasonal Creep of Silty Clay Soil. *Quarterly Journal of Engineering Geology* **8,** 1–29 (1975).
20. Eyles, R. J. & Ho, R. Soil Creep on a Humid Tropical Slope. *Journal of Tropical Geography* **31,** 40–42 (1970).
21. Matsuoka, N. The Relationship between Frost Heave and Downslope Soil Movement: Field Measurements in the Japanese Alps. *Permafrost and Periglacial Processes* **9,** 121–133 (1998).
22. Roering, J. J. Soil Creep and Convex-Upward Velocity Profiles: Theoretical and Experimental Investigation of Disturbance-Driven Sediment Transport on Hillslopes. *Earth Surface Processes and Landforms: The Journal of the British Geomorphological Research Group* **29,** 1597–1612 (2004).
23. Harris, C. Engineering Properties, Groundwater Conditions, and the Nature of Soil Movement on a Solifluction Slope in North Norway. *Quarterly Journal of Engineering Geology* **10,** 27–43 (1977).

24. Boulton, G. S. & Dobbie, K. E. Slow Flow of Granular Aggregates: The Deformation of Sediments beneath Glaciers. *Philosophical Transactions of the Royal Society A: Mathematical, Physical and Engineering Sciences* **356,** 2713–2745 (1998).

25. Clarke, M. F., Williams, M. a. J. & Stokes, T. Soil Creep: Problems Raised by a 23 Year Study in Australia. en. *Earth Surface Processes and Landforms* **24,** 151–175 (1999).

26. Ferdowsi, B., Ortiz, C. P. & Jerolmack, D. J. Glassy Dynamics of Landscape Evolution. *Proceedings of the National Academy of Sciences* **115,** 4827–4832 (2018).

27. Chattoraj, J. & Lemaître, A. Elastic Signature of Flow Events in Supercooled Liquids Under Shear. *Physical Review Letters* **111,** 066001 (2013).

28. Chikkadi, V., Wegdam, G., Bonn, D., Nienhuis, B. & Schall, P. Long-Range Strain Correlations in Sheared Colloidal Glasses. *Physical Review Letters* **107,** 198303 (2011).

29. Jensen, K. E., Weitz, D. A. & Spaepen, F. Local Shear Transformations in Deformed and Quiescent Hard-Sphere Colloidal Glasses. en. *Physical Review E* **90,** 042305 (2014).

30. Desmond, K. W. & Weeks, E. R. Measurement of Stress Redistribution in Flowing Emulsions. *Physical Review Letters* **115,** 098302 (2015).

31. Nicolas, A., Rottler, J. & Barrat, J.-L. Spatiotemporal Correlations between Plastic Events in the Shear Flow of Athermal Amorphous Solids. en. *The European Physical Journal E* **37,** 50 (2014).
