# Supplementary material for: The perpetual fragility of creeping hillslopes
Source: Nat Commun. 2021 Jun 23;12:3909. doi: 10.1038/s41467-021-23979-z (PMC8222271; doi:10.1038/s41467-021-23979-z)
Supplement: Supplementary file 3 — Description of Additional Supplementary Files [file 41467_2021_23979_MOESM3_ESM.pdf]

Description of Additional Supplementary Files

File name: Supplementary Movie 1
Description: Creep strain immediately after preparation Left) Spatial map of creep strain rate across the sandpile immediately after preparation. Right) Time-series of strain rate - red circle indicates timestep of map rendered on left.

File name: Supplementary Movie 2
Description: Creep strain at t = 1024s Left) Spatial map of creep strain across the sandpile for t = 8192s. Right) Evolution of the correlation function for t = 8192s - red circle indicates timestep of map rendered on left.

File name: Supplementary Movie 3
Description: Creep strain at t = 8192s Left) Spatial map of creep strain across the sandpile for t = 1024s. Right) Evolution of the correlation function for t = 1024s - red circle indicates timestep of map rendered on left.

File name: Supplementary Movie 4
Description: Creep strain 11 days after preparation Left) Spatial map of creep strain across the sandpile 11 days (106 s) after preparation. Right) Evolution of the correlation function for 11 days - red circle indicates timestep of map rendered on left.
